# Remits, roles and working models for trial steering committees and data monitoring committees in studies evaluating diagnostic tests: a survey of current practice

**DOI:** 10.1186/1745-6215-14-S1-O29

**Published:** 2013-11-29

**Authors:** Lavinia Ferrante di Ruffano, Jac Dinnes, Jane Daniels, Richard Riley, Nick Hicks, Richard Kaplan, Jon Deeks

**Affiliations:** 1 University of Birmingham, Birmingham, UK; 2 University of Southampton, Southampton, UK; 3 MRC Clinical Trials Unit Hub, London, UK

## Background

All clinical trials that involve the prospective study of patients need some form of independent monitoring. For RCTs of therapeutic interventions, UK public funders of research – including the MRC – require an independent trial steering committee (TSC) to oversee management and finances, and a data monitoring committee (DMC) to oversee study progress and accumulating study data. This fully independent TSC/DMC model may not always be necessary for trials of tests. To address the deficit in guidance regarding oversight arrangements, a project funded by the MRC Midlands Hub for Trials Methodology Research is being undertaken to investigate how DMCs and TSCs should operate.

## Aims and objectives

To develop guidance on how to select between possible working models of TSCs and DMCs when organising oversight procedures for trials of tests.

## Methods

A review of existing guidance regarding the use of TSCs and DMCs for trials of tests, used/published by funding organisations around the world was carried out. A web–based survey and structured interviews of trialists were conducted to establish the breadth of current practice for management and data monitoring during trials of tests.

## Results

We will provide a characterisation of how different TSC and DMC models operate, their roles and responsibilities, why particular models are selected, how models differ according to study design, and which aspects of the chosen working models were particularly useful/disadvantageous.

## Conclusions

Analysis of the review and survey are used to develop insights into how particular models of TSC/DMC should be selected when embarking upon trials of tests.

